# Human eosinophils exert antitumorigenic effects on chordoma

**DOI:** 10.1186/s41016-025-00414-6

**Published:** 2025-12-12

**Authors:** Wang Ying, Li Peng, Bai Jingqiao, Xu Lingzhi, Ren Yuan, Liu Pinan, Wang Bo

**Affiliations:** 1 Beijing Neurosurgical Institute, Capital Medical University, Beijing, China; 2 Department of Neurosurgery, Beijing Tiantan Hospital, Capital Medical University, Beijing, China; 3 Clinical Diagnosis Department of Beijing Tiantan Hospital, Capital Medical University, Beijing, China; 4 Shandong Second Medical University, Weifang, China; 5 Department of Neurosurgery, Beijing Tsinghua Changgung Hospital, School of Clinical Medicine,Tsinghua University, Beijing, China; 6 Department of Neurosurgery, Beijing Neurosurgical Institute, Beijing Tiantan Hospital, Capital Medical University, No.119 South Fourth Ring West Road, Beijing, 100070 P. R. China

**Keywords:** Chordoma, Eosinophil, Apoptosis, TNFα

## Abstract

**Background:**

Chordoma is a devastating rare tumor with a poor prognosis, limited therapeutic options and a high recurrence rate. The exploration of novel therapeutic targets has important clinical significance in chordoma diagnosis, treatment, and outcome prediction.

**Methods:**

In this study, chordoma patients with histopathologically verified disease and KI67 proliferation index data were enrolled. The peripheral eosinophil counts of chordoma patients were summarized, the antitumor effects of eosinophils against chordoma cells were investigated using a coculture experiment, and the potential mechanisms were analyzed.

**Results:**

The chordoma patients were classified into two groups according to KI67 proliferation index: 1) ≤ 5% (*n* = 62), and 2) > 5% (*n* = 80). The results showed that peripheral eosinophil and tumor-infiltrated eosinophil counts decreased with increased KI67 proliferation index, peripheral eosinophil counts deceased after tumor recurrence, and eosinophils could inhibit chordoma cells proliferation by inducing apoptosis and secreting inflammatory cytokines (TNF-α, IL-2 and IFN-γ); moreover, this apoptotic effect could be reversed by blocking TNF-α.

**Conclusions:**

The current study suggests that eosinophils may be a new target for immunotherapy against chordoma.

**Supplementary Information:**

The online version contains supplementary material available at 10.1186/s41016-025-00414-6.

## Background

Chordoma is a low-grade, slow-growing, but locally invasive and aggressive tumor which is characterized by frequent local recurrence [1, 2]. These tumors are resistant to traditional radio/chemotherapy, and surgery is not always effective and is often accompanied by damage to adjacent structures [3, 4]. Thus, chordoma patients have a poor prognosis [5, 6], and new effective targets for chordoma therapy are urgently needed to improve the treatment effects and prolong patient survival.

Tumor immunotherapy is currently a developing approach for the clinical treatment of cancer and accelerates the development of anti-cancer clinical treatment [7–10]. Previous studies have demonstrated that chordomas have extensive interactions with the immune system [11–13], suggesting that immunotherapy might have efficacy in treating chordoma.

The tumor microenvironment (TME) is one of the key factors in tumor immunotherapy, consisting of diverse cell populations in a complex matrix. Increasing evidence suggests that the TME is crucial in the processes of tumor development, metastasis, and drug resistance [14–16]. Eosinophils are an important component of the tumor immune microenvironment, and they can interact with other immune cells to regulate immune responses. They can produce various cytokines and mediators, such as interleukin-4 (IL-4), interleukin-13 (IL-13), etc., to facilitate the differentiation and functional regulation of immune cells. Current researches show that the role of eosinophils in tumors is complex and diverse, with the potential for anti-tumor immunity as well as possible promoting effects on tumor development. However, the specific mechanisms and influencing factors of their actions still require further study and understanding. However, there have been no reports on the study of eosinophils in chordoma thus far [17–19].

In this study, we categorized the chordoma patients into two groups based on the KI67 index, compared the eosinophil counts in these two groups, and examined the impact of eosinophils on the proliferation of chordoma cells and explored the potential molecular mechanisms involved. Therefore, the completion of this study will contribute to further understanding the immune microenvironment of chordoma and exploring the potential of immunotherapy for chordoma treatment. This can provide new insights into the treatment of chordoma from an immunological perspective.

##  Methods

### Chordoma Patients

Chordoma patients with histopathologically verified disease and KI67 proliferation index at Beijing Tiantan Hospital between Dec 2016 and Dec 2019 were analyzed. A total of 142 chordoma cases meeting the inclusion criteria were chosen for the following study. The Institutional Review Board of Beijing Tiantan Hospital approved our study protocols.

### Histology

Tumor samples were fixed in 10% neutral buffered formalin, dehydrated, and embeddedin paraffin. The blocks were then cut into 5 μm thickness and stained with hematoxylin and eosin (HE) for histopathology under light microscope. After HE staining, the eosinophils appear rod-shaped or lobulated, with a deep purple color. The cytoplasm is filled with bright eosinophilic granules that appear red after HE staining.

### Immunohistochemistry (IHC)

Fresh tumor tissues were fixed in 4% paraformaldehyde, dehydrated in ethanol, embedded in paraffin, sectioned at 5μm thickness, and then subjected to deparaffinization, hydration, antigen retrieval (EDTA, PH9.0) and peroxide treatment. Following this, KI67 antibody (ZSGB-BIO, ZM-0166, working solution) were incubated at 4°C overnight, washed with PBS (2 min, 3 times), incubated with secondary antibody for 20 min, washed with PBS (2 min, 3 times), and treated with DAB solution. Finally, tissues were counterstained with hematoxylin and dehydrated. A light microscope was used (Olympus Corporation, Tokyo, Japan).

### Cell lines

UCH1 cells were grown in DMEM (Invitrogen) with the addition of 10% FBS (Invitrogen). The cells were kept in a humidified incubator set to 37°C with 5% CO_2_.

### The eosinophil counts

Total eosinophils were measured through complete blood counts prior to surgery. The absolute eosinophil counts were determined by multiplying the percentage of each component by the total number of WBCs.

### Isolation of eosinophils

Eosinophils were purified as previously described [20]. After anticoagulated peripheral blood was treated with red blood cell lysis buffer to lyse red blood cells, 1 × PBS (containing 2% fetal bovine serum) was used to wash and remove the supernatant. Then, a buffer solution of 1 mL was added and mixed well, followed by the addition of diluted fMLP to achieve a final concentration of 10 nmol/L fMLP, and incubated in a 37°C water bath for 10 min. A 5 mL glass tube was taken, and 0.2 mL of fetal bovine serum was used to wet the tube, followed by the addition of 2 mL of 75% Percoll. Using a pipette, 2 mL of 65% Percoll was slowly added along the wall of the tube to ensure a clear interface between the two layers of Percoll. The fMLP-treated cell suspension was then slowly added along the wall of the tube. The centrifugation was performed at room temperature at 2400 g for 20 min. After centrifugation, a white cloudy layer appeared at the interface between the 75% and 65% Percoll separating solutions, which was the eosinophil layer. The mononuclear cell layer above the 65% Percoll was first aspirated, and then the eosinophil layer was carefully collected and transferred to a 2 mL centrifuge tube. It was washed with PBS and centrifuged at 200 g for 8 min, with the supernatant discarded, and this wash step was repeated three times.

### Suppression assay

UCH1 cells were stained with 1.5 μM CFSE (Molecular Probe/Invitrogen) and then incubated with eosinophils at an E:T ratio of 25:1 ~ 2:1 for 2 ~ 6 h. Flow cytometry was used to measure the proliferation of UCH1 cells based on CFSE dilution.

### Apoptosis analysis

Annexin V-FITC/7-AAD labelling was performed to determine apoptosis in UCH1 cells according to the manufacturer’s recommendations (BD, CA, USA). After 6 h of incubation with eosinophils at an E:T ratio of 25:1 ~ 10:1, UCH1 cells were analyzed by flow cytometry (BD, CA, USA) to determine the percentage of apoptotic cells.

### Cytokine assays

Eosinophil cells were co-incubated with UCH1 cells at an E:T ratio ranging from 25:1 to 10:1 for 6 h, after which the supernatants were collected. A 27-plex cytokine panel (Wayen Biotechnologies, Shanghai, China) was employed to measure the cytokine concentrations in the supernatant, following the manufacturer's instructions. The fluorescence-labeled beads were analyzed using a calibrated Bio-Plex MAGPIX system (Bio-Rad, Luminex Corporation, Austin, TX, United States), and the cytokine concentrations were calculated using Bio-Plex Manager 6.1 (Bio-Rad).

#### Statistical analysis

Student's t-test was employed to compare two groups of quantitative variables that followed a normal distribution, with a *p*-value of less than 0.05 considered statistically significant. All data are expressed as the mean ± standard deviation (SD), and all statistical analyses were conducted using Prism 6 software (La Jolla, CA, USA)..

## Results

### Patient information

A total of 142 patients with pathologically confirmed skull base chordoma, along with KI67 proliferation index data, treated in Beijing Tiantan Hospital between Dec 2016 and Dec 2019 were selected for the present study. Among the 142 chordoma patients, 90 (63.38%) patients were male, 52 patients (36.62%) were female, with a mean age of 40.48 years (SD ± 18.42 years), and 29 patients (20.42%) had tumor recurrence during the statistical period. The clinical data are summarized in Table 1.

**Table 1:**
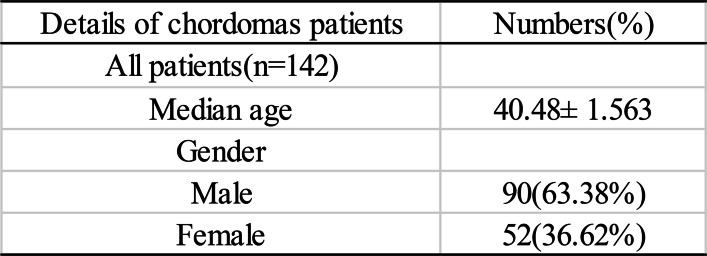
Details of Chordoma Patients

### Eosinophil counts correlated with chordoma proliferation rates

**Fig. 1:**
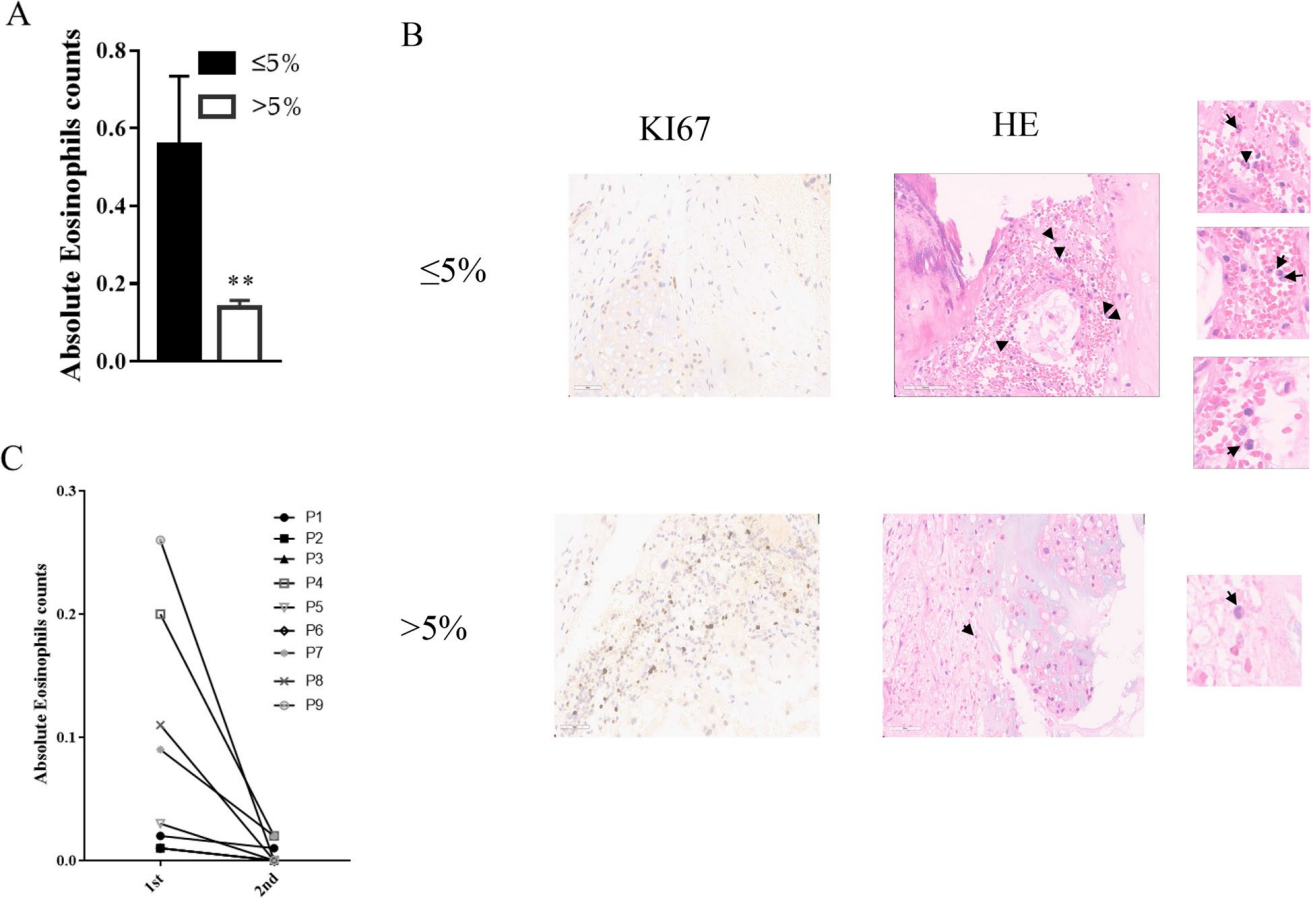
EOS counts are correlated with chordoma cell proliferation rates. **A** Peripheral eosinophil counts decreased with increasing KI67 proliferation index. **B** Tumor-infiltrated eosinophil counts decreased with increasing KI67 proliferation index. **C** Peripheral eosinophil counts were reduced after tumor recurrence. The data are presented as the mean ± SD. *p < 0.05

The chordoma patients were classified into two groups according to KI67 proliferation index: 1) ≤ 5% (*n* = 62), and 2) > 5% (*n* = 80). Further study showed that the peripheral eosinophil counts of these two groups were as follows: 1) 0.5578 ± 1.3440(KI67 ≤ 5%); 2) 0.1382 ± 0.1651(KI67 > 5%) (Mean ± SD), suggesting that peripheral eosinophil counts decreased with the increases in KI67 proliferation index (Fig. 1A) and that the number of tumor-infiltrated eosinophils had the same trend (Fig. 1B). Further investigation also showed that the peripheral eosinophil counts were reduced in 9 patients (9/29, 31.03%) after tumor recurrence (Fig. 1C), suggesting that eosinophils may play a role in the proliferation of chordoma.

### Human eosinophils inhibit chordoma cell line proliferation

**Fig. 2:**
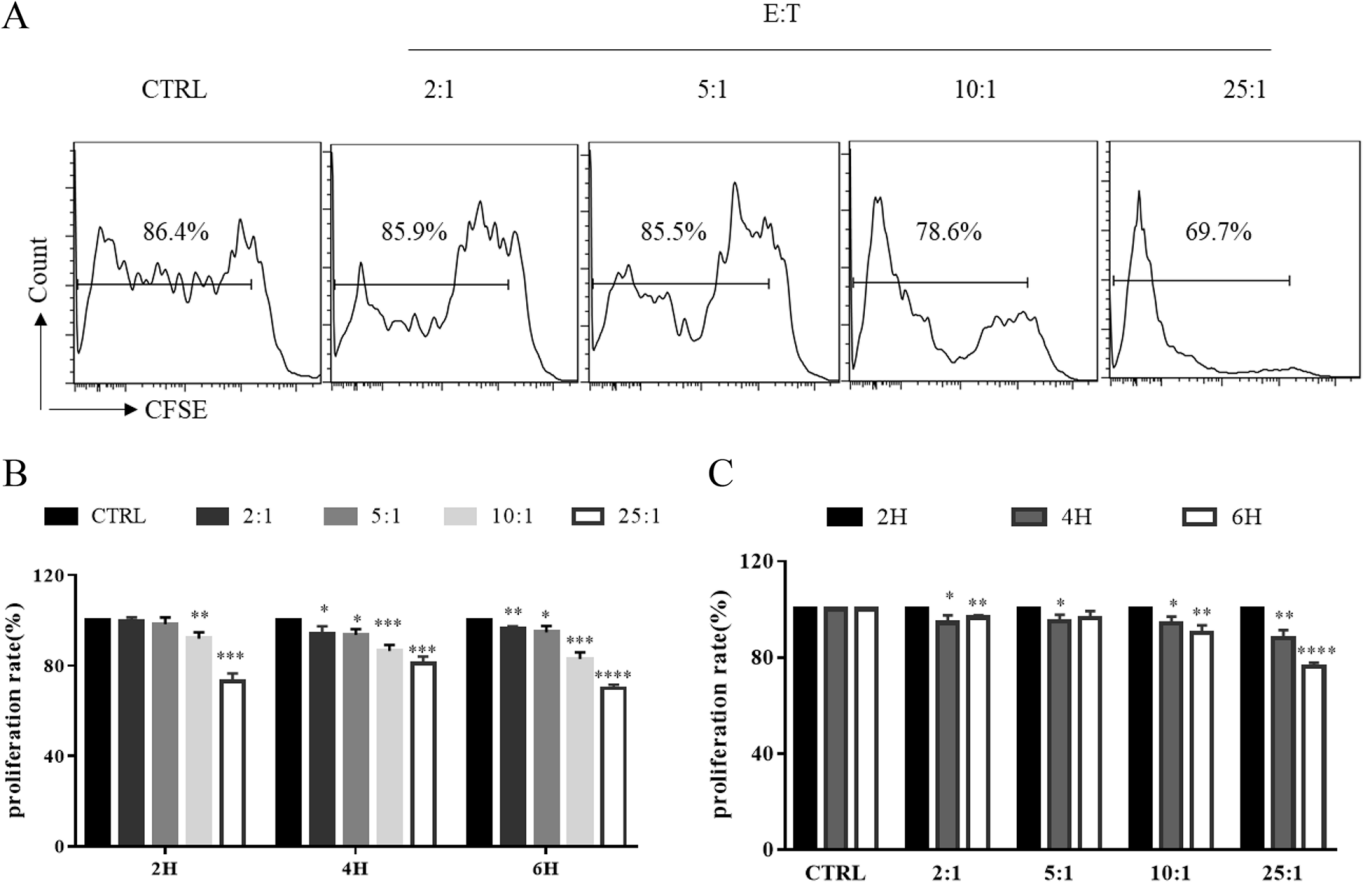
Human eosinophils inhibit the proliferation of a chordoma cell line. **A** Cytometric analysis of the proliferation of UCH1 cells after coculture with eosinophils at an E:T ratio of 25:1 ~ 2:1 for 4 h. **B** Statistical analysis of UCH1 cell proliferation after coculture with eosinophils at an E:T ratio of 25:1 ~ 2:1 for 2 ~ 6 h. Data are expressed as mean ± SD. *p < 0.05, **p < 0.01, ***p < 0.005, ****p < 0.001, compared to the control group

Next, we investigated the antitumoral activity of purified human eosinophils towards a chordoma cell line (UCH1). CFSE-labelled UCH1 cells were cocultured with eosinophils at an E:T ratio of 25:1 ~ 2:1 for 2 ~ 6 h, and pure tumor cells were used as a control. The results showed that eosinophils were able to induce UCH1 cell death in a time- and dose-dependent manner (Fig. 2).

### Eosinophils induce chordoma cell line apoptosis

**Fig. 3:**
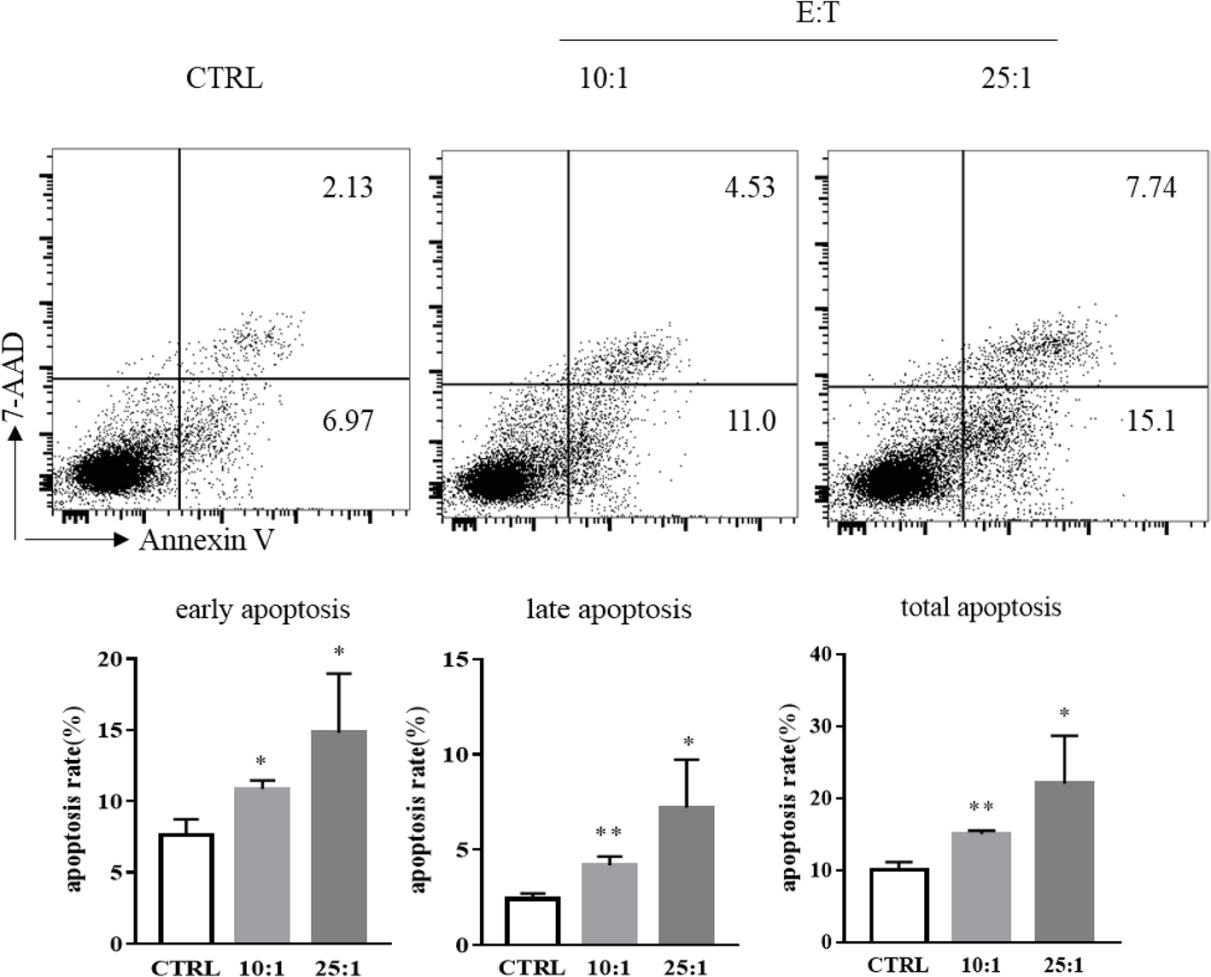
Eosinophils induce the apoptosis of chordoma cell. Upper: Flow cytometric analysis of UCH1 cells after coculture with eosinophils at an E:T ratio of 25:1 ~ 10:1 for 6 h using Annexin V-PE/7-AAD staining. Lower: Statistical analysis of the apoptosis rate of UCH1 cells after coculture with eosinophils at an E:T ratio of 25:1 ~ 10:1 for 6 h. Data are expressed as mean ± SD. *p < 0.05, **p < 0.01, ***p < 0.005, ****p < 0.001, compared to the control group

Subsequently, we evaluated eosinophil-induced apoptosis using an Annexin V/7-AAD staining assay. As shown in Fig. 3, eosinophils induced an apoptosis rate of 22.01 ± 3.847% among total cells after 4 h at an E:T ratio of 25:1 and 15.04 ± 0.280% at an E:T ratio of 10:1, and 10.04 ± 1.106% in tumor cells only. Our finding indicated that the eosinophils had a dose-dependent influence on the programmed cell death of chordoma cells.

### The proposed mechanism by which human eosinophils inhibit chordoma cells proliferation

**Fig. 4:**
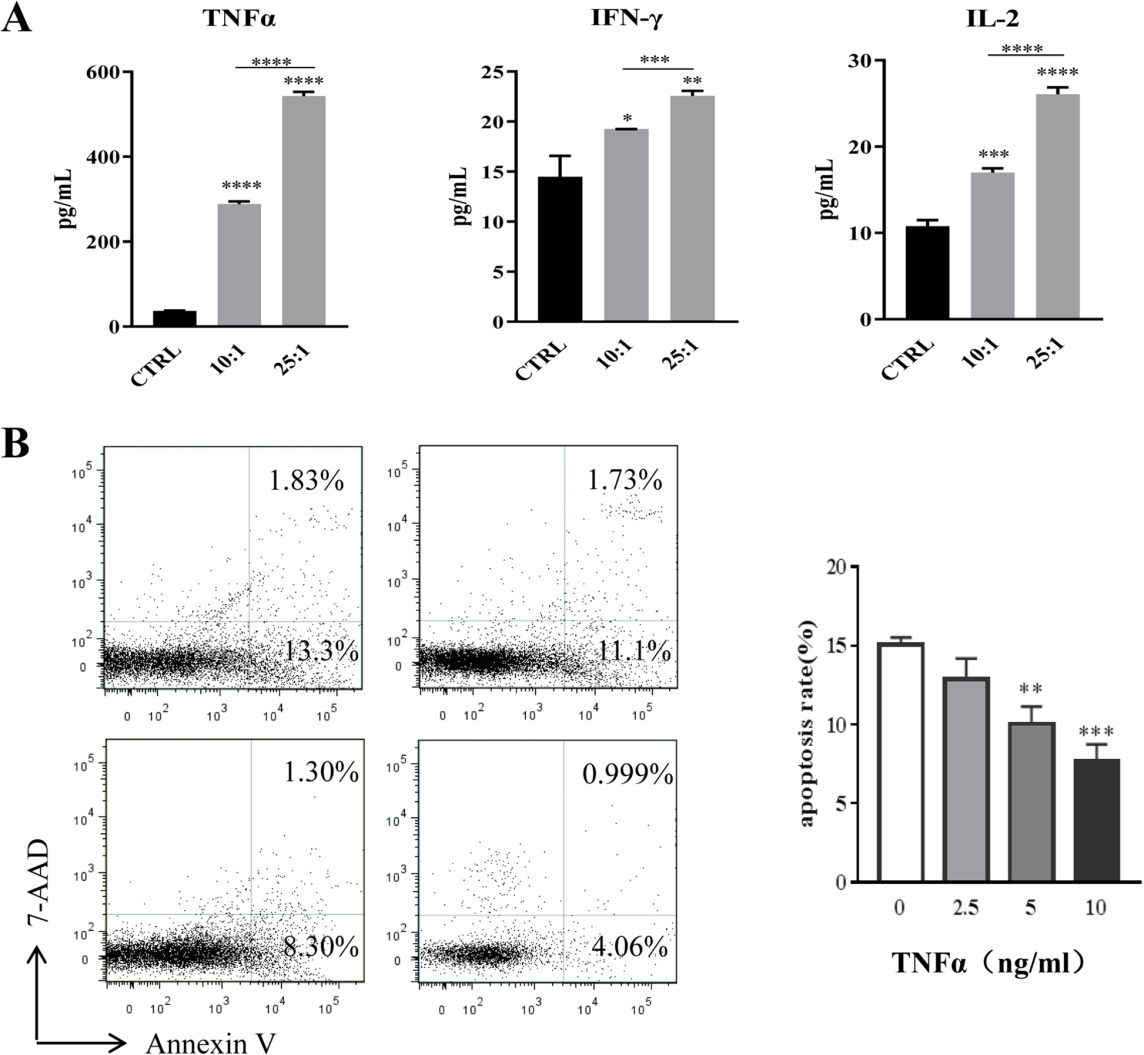
The proposed mechanism by which human eosinophils inhibit chordoma cell proliferation. **A** TNF-α, IL-2 and IFN-gamma increased significantly with increasing E:T ratios. **B** The apoptosis rate of chordoma cells induced by eosinophils could be partly inhibited by blocking TNF-α. Data are expressed as mean ± SD. *p < 0.05, **p < 0.01, ***p < 0.005, ****p < 0.001, compared to the control group

We further examined the cytokine secretion profiles of the coculture supernatant. As shown in Fig. 4A, TNF-α, IL-2 and IFN-γ increased significantly with increasing E:T ratios. IL-6, IL-8 and IL-10 were not significantly different (*p* > 0.05, data not shown). PDGF-BB, VEGF, IL-1β, IL-1RA, G-CSF, RANTES, MIP-1α, and eotaxin also increased significantly with increasing E:T ratios (Fig. S1). This cytotoxic effect of eosinophils on chordoma cells could be inhibited by blocking TNF-α via neutralizing antibodies (Certolizumab pegol, MCE, HY-P9953), which resulted in a 22% decrease in apoptosis rate at 5 μg/ml and 41% decrease at 10 μg/ml (Fig. 4B).

## Discussion

In this study, we classified the chordoma patients into two groups according to KI67 proliferation index: 1) ≤ 5% (*n* = 62), and 2) > 5% (*n* = 80). Further study showed that peripheral eosinophil counts decreased with increases in KI67 proliferation index and that the number of tumor-infiltrated eosinophils had the same trend. Next, we evaluated the antitumor effects of eosinophils on chordoma cells, and the results showed that eosinophils could inhibit chordoma cell proliferation by inducing apoptosis and secreting inflammatory cytokines (TNF-α, IL-2 and IFN-γ), and the apoptotic effects could be reversed by blocking TNF-α via neutralizing antibodies, suggesting that eosinophils could inhibit chordoma cell proliferation by inducing apoptosis, and TNF-α may play a role in chordoma cell death induced by eosinophils.

Ki-67 is a protein marker used to measure the proliferation or growth rate of cells. It is commonly used as a biomarker in cancer research and clinical practice to assess the level of cell division within a tumor. A high Ki-67 index typically indicates more actively dividing cells and is associated with a more aggressive tumor phenotype. Previously studies have reported that chordomas with a KI67 proliferation index over 5% had a shorter doubling time and/or shorter overall survival, which may be an unfavorable prognostic marker [21–23]. Referring to the above, in the current study, we divided 142 chordoma patients into two groups according to the KI67 proliferation index as follows: 1) ≤ 5%, and 2) > 5%. The results showed that the chordoma patients with a higher KI67 index (KI67 > 5%) had lower peripheral eosinophil counts and fewer tumor-infiltrated eosinophils. The above results suggest that eosinophils may play a role in the occurrence and development of chordoma.

Eosinophils are multifunctional granulocytes that are related to the modulation of different inflammatory responses, including helminth infections, allergic diseases and tumor immunity [24–26]. Although their role in some tumors remains unclear, increasing data have indicated that eosinophils exert antitumor effects by releasing cytotoxic granules, cytokines and chemokines. Furthermore, eosinophils can modulate antitumor immunity by interacting with other immune cells, including macrophages, DCs and T cells [19, 27–29]. In this study, the results showed that both peripheral and tumor-infiltrated eosinophils decreased with the increases in KI67 proliferation index. Further investigation also showed that the peripheral eosinophil counts were reduced in 9 patients (9/29, 31.03%) after tumor recurrence, indicating that eosinophils may related to chordoma proliferation. Further research showed that eosinophils could inhibit chordoma cell proliferation by inducing apoptosis and secreting inflammatory cytokines, and the apoptotic effects could be reversed by blocking with anti-TNFα Ab, further confirming the antitumor role of eosinophils.

## Conclusions

In summary, this study demonstrated that eosinophils in chordoma may exert an inhibitory effect on tumor proliferation by secreting the inflammatory cytokine TNF-α, which promotes tumor cell apoptosis. This article starts from the perspective of eosinophils in the immune microenvironment and provides evidence for their inhibitory role in the proliferation of chordoma. It explores the possible mechanisms involved, offering a new therapeutic approach for the treatment of chordoma.

## Supplementary Information

Supplementary Material 1: Figure S1. PDGF-BB, VEGF, IL-1β, IL-1RA, G-CSF, RANTES, MIP-1α, and eotaxin increased significantly with increasing E:T ratios.

## Data Availability

Data sharing is not applicable to this article as no new data were created or analyzed in this study.
