# Supplementary figures and images for: Human eosinophils exert antitumorigenic effects on chordoma
Source: Chin Neurosurg J. 2025 Dec 12;11:32. doi: 10.1186/s41016-025-00414-6 (PMC12699895; doi:10.1186/s41016-025-00414-6)

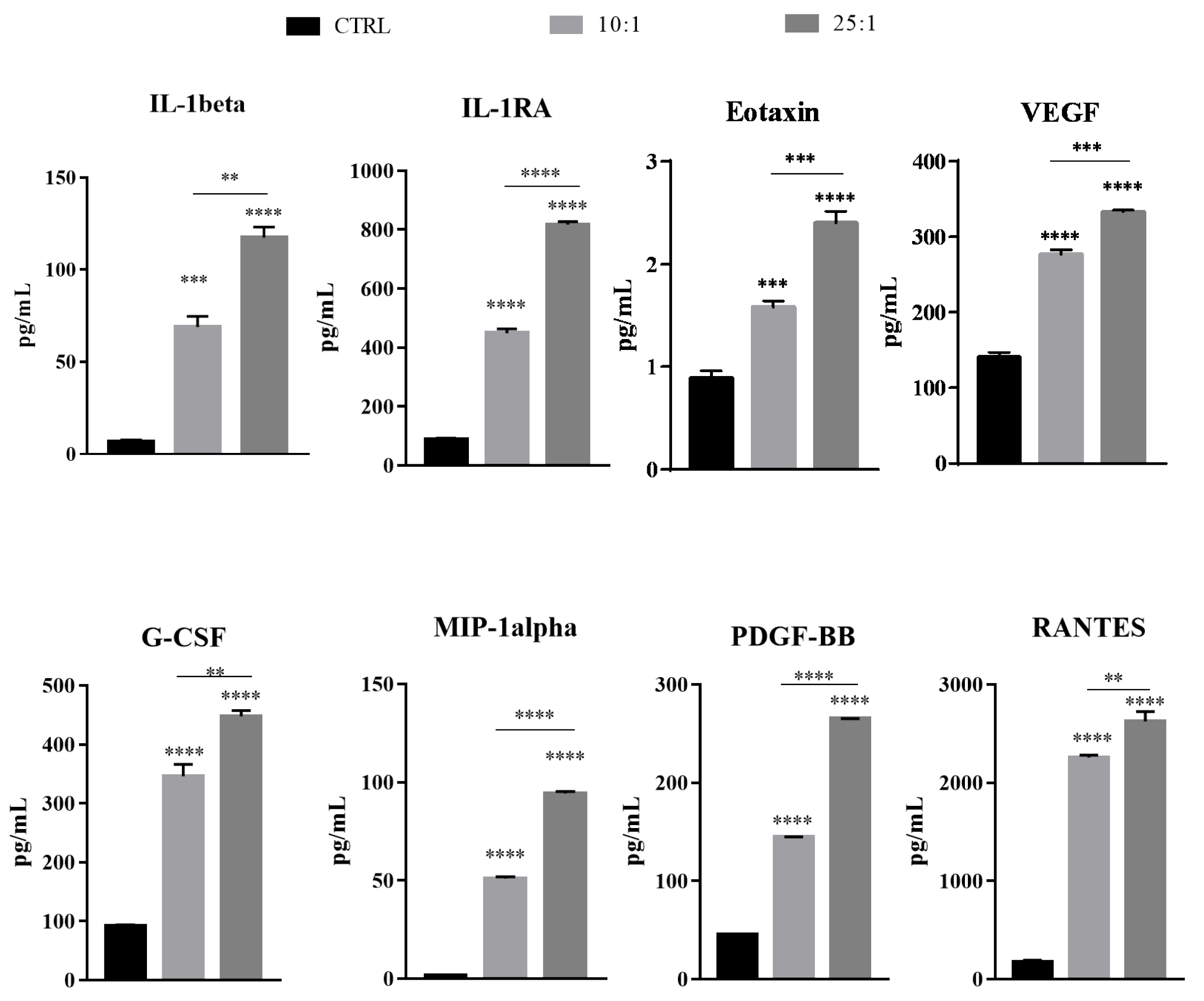

Supplement: Supplementary file 1 — Supplementary Material 1: Figure S1. PDGF-BB, VEGF, IL-1β, IL-1RA, G-CSF, RANTES, MIP-1α, and eotaxin increased significantly with increasing E:T ratios. [file 41016_2025_414_MOESM1_ESM.tif]
